# Modified Rice Bran Dietary Fiber-Based Pre-Emulsion as a Fat Replacer: Modulating Physicochemical and Sensory Properties of Emulsified Meat Gels

**DOI:** 10.3390/foods15111929

**Published:** 2026-05-29

**Authors:** Yuhui Zhao, Chu Zhang, Xue Zhao, Xinglian Xu

**Affiliations:** State Key Laboratory of Meat Quality Control and Cultured Meat Development, Key Laboratory of Meat Processing, Ministry of Agriculture, Jiangsu Synergetic Innovation Center of Meat Production and Processing, College of Food Science and Technology, Nanjing Agricultural University, Nanjing 210095, China

**Keywords:** rice bran dietary fiber, fat replacer, emulsified meat, rheology, dynamic sensory evaluation

## Abstract

Polysaccharide-based pre-emulsions offer a promising strategy for reducing saturated fat in emulsified meat products. In this study, a pre-emulsion stabilized by rice bran dietary fiber modified with alkaline hydrogen peroxide (MRF) was used to replace pork back fat in emulsified meat gels. Four model systems were prepared, varying in fat content (20% and 50%) and chopping intensity (low vs. high). MRF pre-emulsion significantly reduced fat globule size (e.g., D_[4,3]_ decreased by 18–34%, D_[3,2]_ by up to 83%) and improved shear stability, as reflected in the weaker frequency dependence of the storage modulus (G′). In high-chopping systems, MRF substitution increased gel elasticity but lowered hardness (by 25–30%), chewiness, and shear force (by 20–25%). Low-field NMR analysis revealed a partial shift from immobilized to free water, which raised cooking loss by 2–4 percentage points while enhancing perceived juiciness. Color measurements indicated that MRF effectively offset the loss of lightness typically associated with fat reduction. Both quantitative descriptive analysis (QDA) and temporal dominance of sensations (TDS) confirmed that MRF-substituted samples showed a markedly lower dominance of fatty sensation during the late oral processing stage (30–40% reduction in dominance rate), whereas the overall dynamic sensory profile remained similar to that of full-fat controls. Collectively, these results demonstrate that MRF, as a functional polysaccharide, stabilizes the system through hydration-induced swelling, hydrogen bonding with myofibrillar proteins, and the formation of a composite interfacial film around fat globules. These mechanisms enhance emulsion stability and successfully mimic the oral textural properties of animal fat, supporting the use of MRF as an effective polysaccharide-based fat replacer in reduced-fat meat products.

## 1. Introduction

Emulsified meat products, including sausages, meatballs, and patties, are gel-type emulsified foods manufactured by chopping and cooking lean meat together with animal fat. Their distinctive texture and flavor account for their widespread popularity [1,2]. Animal fat, however, contains high levels of saturated fatty acids (SFAs), and excessive consumption is associated with an elevated risk of chronic conditions such as cardiovascular disease, diabetes, and obesity. Consequently, interest in low-fat and health-oriented diets has grown substantially [3]. Because animal fat is integral to the quality of emulsified meat products, fat-reduced or defatted versions frequently lack the mouthfeel and flavor of traditional counterparts. Developing fat replacers that lower fat content without compromising sensory quality has therefore become a research priority [4].

Vegetable oils are rich in unsaturated fatty acids, but their direct substitution for animal fat often causes water and oil exudation as well as oxidative deterioration [5]. Pre-emulsification can stabilize vegetable oils by promoting their uniform dispersion within the meat gel matrix, thereby enhancing product stability [6]. Among potential stabilizers, dietary fiber (DF), a functional polysaccharide, has emerged as an attractive option due to its low caloric value, high water-binding capacity, and favorable effects on gut microbiota. Recent advances have further highlighted the potential of polysaccharide-based fat replacers in developing healthier meat products [7,8]. Mechanistically, DF absorbs water and swells, which increases the viscosity of the continuous phase. Its abundant hydroxyl groups also enable hydrogen bonding with myofibrillar proteins, facilitating the development of a viscoelastic network. Furthermore, DF can adsorb at the oil–water interface and co-stabilize fat globules through steric hindrance and electrostatic repulsion, thereby improving emulsion stability [9]. A growing body of research indicates that incorporating dietary fiber into pre-emulsions as a partial substitute for animal fat helps preserve both the functional performance and the eating quality of emulsified meat products [10,11]. Among various DFs, both soluble and insoluble types have been explored as fat replacers, each with distinct functional characteristics. Soluble fibers, such as β-glucan, inulin, and psyllium, exhibit strong water-binding capacity and contribute to viscosity and gel formation, making them suitable for mimicking the creamy texture of fat in emulsified systems [12]. However, their rheological behavior under heating differs from the melting behavior of animal fat. Insoluble fibers, by contrast, retain their particulate structure and can serve as active or inactive fillers that reinforce the gel matrix through water/oil absorption and non-covalent interactions, partially compensating for the structural loss upon fat reduction [13]. Nevertheless, high levels of insoluble fibers may lead to a gritty or coarse mouthfeel due to their inherent particle characteristics. Rice bran, a major agricultural byproduct, represents a high-quality source of DF. Treatment with alkaline hydrogen peroxide markedly improves its water-holding and emulsifying properties by increasing the exposure of hydrophilic groups (such as carboxyl and hydroxyl) along the polysaccharide chains and by partially converting insoluble fiber into soluble fragments, which enhances both hydration capacity and interfacial activity [14]. Thus, preparing pre-emulsions with modified rice bran dietary fiber (MRF) for fat replacement not only improves the nutritional profile of meat products but also offers a promising route for the high-value utilization of processing byproducts.

Although considerable progress has been made in reducing fat in emulsified meat products using pre-emulsion technology, most studies to date have examined the effects of fat replacers on initial physicochemical properties within relatively simple, single-matrix systems [15,16]. The structural complexity of emulsified meat products as multiphase systems has received far less attention. Moreover, current research on polysaccharide-based fat replacers has primarily focused on in vitro characterization, such as rheology and texture, with limited integration of dynamic sensory perception during oral processing. As highlighted in recent reviews, there remain critical knowledge gaps regarding how emulsion gel-based fat replacers regulate oral lubricity, flavor perception, and dynamic digestive behaviors under realistic consumption conditions [17,18]. Sensory perception in these products is not governed by any single component but rather emerges from the interplay between formulation composition and processing conditions. Fat content and chopping intensity are key parameters that govern product properties: fat content determines fat globule size/distribution, protein network continuity, and lipid release [19]; chopping intensity controls protein extraction, fat particle reduction, and interfacial film formation [20]. Their interplay is especially critical for polysaccharide-based emulsion fat replacers, as the hydration and interfacial behavior of the polysaccharide depend on mechanical energy and matrix composition. Building on earlier work showing that a pre-emulsion prepared with MRF and soybean oil can preserve the quality of emulsified meat products [21], the present study employs this MRF pre-emulsion as a fat replacer. Four model systems were constructed to reflect common commercial product types, varying in fat content (20% and 50%) and chopping intensity (low and high), and fat substitution was carried out to produce emulsified meat gels. The study examines both physicochemical and sensory properties of the resulting gels. Measurements included rheological behavior, particle size distribution, cooking loss, shear force, and texture profile of the meat batters and gels. In parallel, static sensory profiling (quantitative descriptive analysis, QDA) and dynamic sensory evaluation (temporal dominance of sensations, TDS) were conducted to characterize changes in sensory perception during oral processing, thereby establishing a foundation for future investigations of oral processing behavior.

## 2. Materials and Methods

### 2.1. Materials

Natural rice bran dietary fiber was purchased from Shaanxi Mufei Biotechnology Co., Ltd (Xi‘an, Shannxi, China). According to our measurement, the modified rice bran dietary fiber (MRF) contained 63.56% total dietary fiber, 2.33% protein, and 1.26% fat. Chilled chicken breast (containing 23.90% protein and 0.33%) was obtained from Tyson Foods Co., Ltd (Springdale, Arkansas, USA). Fresh pork back fat (containing 84.72% fat), Jinlongyu soybean oil, and seasonings were sourced from local markets in Nanjing, China. The chicken breast and pork back fat were obtained from different commercial sources and were not from the same slaughter batch. Hydrogen peroxide, sodium hydroxide, hydrochloric acid, and anhydrous ethanol were supplied by Sinopharm Chemical Reagent Co., Ltd (Shanghai, China). Sodium dodecyl sulfate (SDS) was acquired from Shanghai Aladdin Biochemical Technology Co., Ltd (Shanghai, China). All chemical reagents were of analytical grade or higher.

### 2.2. Construction of Emulsified Meat Product Model Systems

Four model systems representing common emulsified meat products on the Chinese market (including meatballs, lion’s head meatballs, and emulsified sausages) were established by varying two factors: fat content at 20% and 50%, and chopping intensity at low (1500 r/min) or high (3000 r/min). The treatment group designations are summarized in Table 1.

### 2.3. Preparation of MRF and Its Pre-Emulsion

MRF was prepared using the alkaline hydrogen peroxide method described by Wang et al. [21]. In brief, natural rice bran dietary fiber was weighed using an electronic balance (AUY 120, Shimadzu, Kyoto, Japan) and mixed with 2% H_2_O_2_ solution at a solid-to-liquid ratio of 1:20 (*w*/*v*). The mixture was stirred magnetically for 1 h in a water bath (Jiangnan Experimental Instrument Factory, Changzhou, China) to achieve uniform dispersion, after which the pH was adjusted to 11.5 with 1 mol/L NaOH using a benchtop pH meter (FiveEasy Plus, Mettler, Columbus, OH, USA). The suspension was then placed in a 60 °C water bath and stirred magnetically for an additional 6 h. Following the reaction, the pH was neutralized to 7 with 1 mol/L HCl. The mixture was centrifuged at 3000 rpm for 15 min at 4 °C (Avanti J-E, Beckman Coulter, Brea, USA) to collect the precipitate, which was washed twice with anhydrous ethanol at a 1:1 (*w*/*w*) ratio. The washed precipitate was spread evenly and dried in a forced-air oven (BGL-30B, Shanghai Ben Ting Instrument Co., Ltd., Shanghai, China) at 37 °C for 12 h. The dried product was ground and passed through a 120-mesh sieve (Taizhou Yueyang Trading Co., Ltd., Taizhou, Zhejiang, China) to obtain MRF.

To prepare the MRF pre-emulsion, MRF was dispersed in ultrapure water at a ratio of 0.3:10 (*w*/*v*) and hydrated overnight with magnetic stirring. The hydrated MRF dispersion was then mixed with soybean oil at a volume ratio of 4:1 (*v*/*v*). The mixture was homogenized using a high-speed homogenizer (Ultra-Turrax T25, IKA, Staufen, Germany) in an ice bath at 12,000 rpm for 2 min (three 40 s cycles) to obtain the MRF emulsion.

### 2.4. Preparation of Emulsified Meat Batter

The emulsified meat batter was prepared according to the method of Zhang et al. with slight modifications [22]. Chicken breast was trimmed of surface fat and connective tissue, and pork back fat was minced using a meat grinder. Chopping and emulsification were performed in three steps using a chopper (S22-LA363, Joyoung Co., Ltd., Jinan, Shandong, China). First, 40% of the lean meat and pork back fat were placed in the chopper and chopped at the designated speed (1500 or 3000 r/min) for 1 min, while 1/3 of the ice water was added. Ice water was prepared with an ice maker (SIM-F12, Sanyo, Osaka, Japan). Second, the remaining 60% of the lean meat, other ingredients, and a second 1/3 of the ice water were added and chopped at the same speed for 1 min. Finally, the remaining 1/3 of the ice water was added, and the mixture was chopped again at the same speed for 1 min. The chopper was paused for 2 min between steps to prevent overheating, and the core temperature of the mixture was maintained below 10 °C throughout the process. The formulations of the emulsified meat batter samples are listed in Table 2.

For the preparation of emulsified meat gels, the method of Wang et al. was followed with slight modifications [23]. The emulsified meat batter was transferred into 50 mL centrifuge tubes and centrifuged at 500× *g* for 3 min at 4 °C in an ultra-speed refrigerated centrifuge to remove air bubbles. The tubes were then heated in a water bath from 20 °C to 80 °C and held at 80 °C for 20 min. After cooling to 25 °C, emulsified meat gels were obtained.

### 2.5. Rheological Properties

The rheological properties of the emulsified meat batters were measured using a rheometer (MCR302e, Anton Paar, Graz, Austria) equipped with parallel plate geometry (50 mm diameter, 1 mm gap). Freshly prepared meat batter was placed on the plate, excess sample was trimmed, and the sample was allowed to equilibrate for 2 min before measurement. (1) Apparent viscosity: Measurements were performed at a constant temperature of 25 °C over a shear rate range of 0.01 to 1000 s^−1^, and the apparent viscosity was recorded as a function of shear rate. (2) Dynamic frequency sweep: Frequency sweeps were conducted at 25 °C with a strain of 0.1% (within the linear viscoelastic region) over an angular frequency range of 0.1 to 100 rad/s. The storage modulus (G′) and loss modulus (G″) were recorded as functions of angular frequency. (3) Dynamic temperature sweep: Temperature sweeps were performed at a fixed angular frequency of 1 rad/s and a strain of 0.1% (within the linear viscoelastic region). The temperature was increased from 20 °C to 80 °C at a heating rate of 2 °C/min, and changes in G′ and G″ were monitored. The protective cover of the instrument was lowered throughout the test to ensure precise temperature control [24].

### 2.6. Particle Size

The prepared emulsified meat batter was diluted with 0.01% (*w*/*v*) sodium dodecyl sulfate (SDS) solution at a ratio of 1:30 (*w*/*v*). The mixture was homogenized using a high-speed homogenizer (Ultra-Turrax T25, IKA, Staufen, Germany) at 1000 rpm for 20 s and then filtered through two layers of cheesecloth to prevent clogging of the instrument. Droplet size and size distribution of the diluted meat batter were measured using a laser particle size analyzer (Mastersizer 3000, Malvern Panalytical, Malvern, Worcestershire, UK). The following measurement parameters were used: refractive index of the dispersed phase—1.456; refractive index of the dispersant (distilled water)—1.333; absorption index—0.001; laser obscuration range—5% to 15%. Values for D_10_, D_50_, D_90_, D_[3,2]_, D_[4,3]_, as well as particle size distribution curves, were recorded [25].

### 2.7. Cooking Loss

The prepared emulsified meat batter was transferred into 50 mL centrifuge tubes and weighed (W_1_). After heating as described in Section 2.4, the resulting gels were removed from the tubes, gently blotted with filter paper to remove surface moisture, and weighed again (W_2_). Cooking loss was calculated using the following equation (Equation (1)):

(1) $${\mathrm{Cooking}\;\mathrm{loss}\;(\%)\;=\;(W}_{1}{-W}_{2}{)\;/\;W}_{1}\times \;100$$

### 2.8. Low-Field Nuclear Magnetic Resonance (LF-NMR) Water Distribution

Water distribution in the meat gels was determined using an LF-NMR imaging analyzer (PQ001, Niumag Electric Technology Co., Ltd., Shanghai, China). Following the method of Lin et al. [26], the gels were cut into cylindrical samples (25 mm diameter × 15 mm height), wrapped with plastic wrap, and placed into NMR tubes for measurement. The CPMG pulse sequence was applied at a test temperature of 32 °C. Acquired data were inverted using Multiexp Inv Analysis software (Version 4.08) according to the following mathematical model (Equation (2)):

(2) $$A(t)=\sum \left[A_{0i}\exp(\frac{-t}{T_{2i}})\right]$$

where *A*(t) is the amplitude at decay time t (ms), A_0i_ is the equilibrium amplitude of the i-th component, and T_2i_ is the spin-spin relaxation time (ms) of the i-th component.

### 2.9. Color Measurement

Color parameters of the emulsified meat batters and gels were measured using a portable handheld colorimeter (CR-400, Konica Minolta, Osaka, Japan) with Illuminant D65 and a 2° standard observer angle. The batters were spread evenly in a Petri dish, while the gels were cut into cylindrical pieces (25 mm diameter × 5 mm height). Lightness (L*), redness (a*), and yellowness (b*) were recorded. Before each measurement session, the instrument was calibrated against a standard white plate (L* = 96.86, a* = −0.15, b* = 1.87). For each sample (both meat batter and gel), four replicate measurements were performed.

### 2.10. Shear Force

Shear force of the emulsified meat gels (only on the gel samples, not on the raw meat batter) was measured using a muscle tenderness meter (C-LM, College of Engineering, Northeast Agricultural University, Harbin, China) equipped with a Warner–Bratzler shear blade. Gel samples were cut into rectangular strips measuring 1 cm × 1 cm × 3 cm and placed under the blade for shearing. The maximum peak force during shearing was recorded as the shear force.

### 2.11. Texture Profile Analysis (TPA)

Texture properties of the gel samples were analyzed using a texture analyzer (TA-XT Plus, Stable Micro Systems, Godalming, UK) following the method of Bibat et al. with slight modifications [27]. Samples were cut into cylindrical pieces (25 mm diameter × 10 mm height) and placed at the center of the platform. Each sample was compressed twice vertically using a cylindrical probe (P/50, 50 mm diameter), and the texture characteristic curve was recorded. The test parameters were as follows: pre-test speed, 5.0 mm/s; test speed, 5.0 mm/s; post-test speed, 5.0 mm/s; compression strain, 50% of original sample height; trigger force, 5.0 g.

### 2.12. Quantitative Descriptive Analysis (QDA)

Sensory properties of the samples were evaluated using QDA. The panel consisted of 10 assessors (4 males and 6 females, aged 24–29 years) with normal oral health and regular consumption of meat products. Based on preliminary trials, ten attributes with the greatest influence on sensory perception were selected for evaluation: hardness, springiness, chewiness, juiciness, fibrousness, adhesiveness, fattiness, umami, aftertaste, and brightness. Definitions of these attributes and the corresponding reference materials are provided in Table 3. Prior to the evaluation, panelists underwent training to become familiar with the QDA procedure and attribute definitions, ensuring consistent identification and scoring of each attribute.

The evaluation was conducted in a dedicated sensory laboratory. Samples were placed evenly into 50 mL tasting cups and coded with random three-digit numbers. Panelists chewed each sample until swallowing and independently scored the sensory attributes. A minimum interval of 1 min was maintained between samples, and panelists rinsed their mouths with water before and after each test.

### 2.13. Temporal Dominance of Sensations (TDS)

TDS was used to capture dynamic changes in sensory attributes during oral processing of the samples. Based on the QDA results, six representative attributes were selected for evaluation: hardness, smoothness, stickiness, juiciness, meatiness, and fattiness. The panel consisted of 10 assessors (6 females and 4 males, aged 24–29 years) with normal oral health and regular consumption of meat products. Before the formal evaluation, panelists were trained to become familiar with the TDS procedure, software operation, attribute definitions, and attribute recognition. Based on preliminary trials, the evaluation time per sample was set to 24 s.

The evaluation was conducted in a dedicated sensory laboratory. Samples were placed evenly into 50 mL tasting cups and coded with random three-digit numbers. Panelists placed each sample in their mouths, started the timer, and chewed normally. Dominant sensations were recorded in real time using the sensory analysis program APPsense (Version 10.7.0), with panelists selecting the current dominant attribute every 3 s until the 24 s period ended. Results are presented as TDS curves showing the dominance rate of each sensory attribute over time, and significance levels were calculated accordingly.

### 2.14. Statistical Analysis

All experiments were independently replicated at least three times to ensure accuracy and reliability, and results are expressed as means ± standard deviation. Statistical analysis was performed using IBM SPSS Statistics 26 (IBM, Armonk, NY, USA). Two-way analysis of variance (ANOVA) was used to evaluate the effects of formulation (four levels: 20% fat, 20% fat with partial replacement, 50% fat, and 50% fat with partial replacement), chopping intensity (two levels: low and high), and their interaction on the measured parameters. Significant differences among individual treatment groups were determined by one-way ANOVA followed by Duncan’s multiple range test. Differences were considered statistically significant at *p* < 0.05. Graphical visualization was carried out using Origin 2026 (OriginLab, Northampton, MA, USA).

## 3. Results

### 3.1. Properties of Emulsified Meat Batters and Gels

#### 3.1.1. Rheological Properties

**Figure 1:**
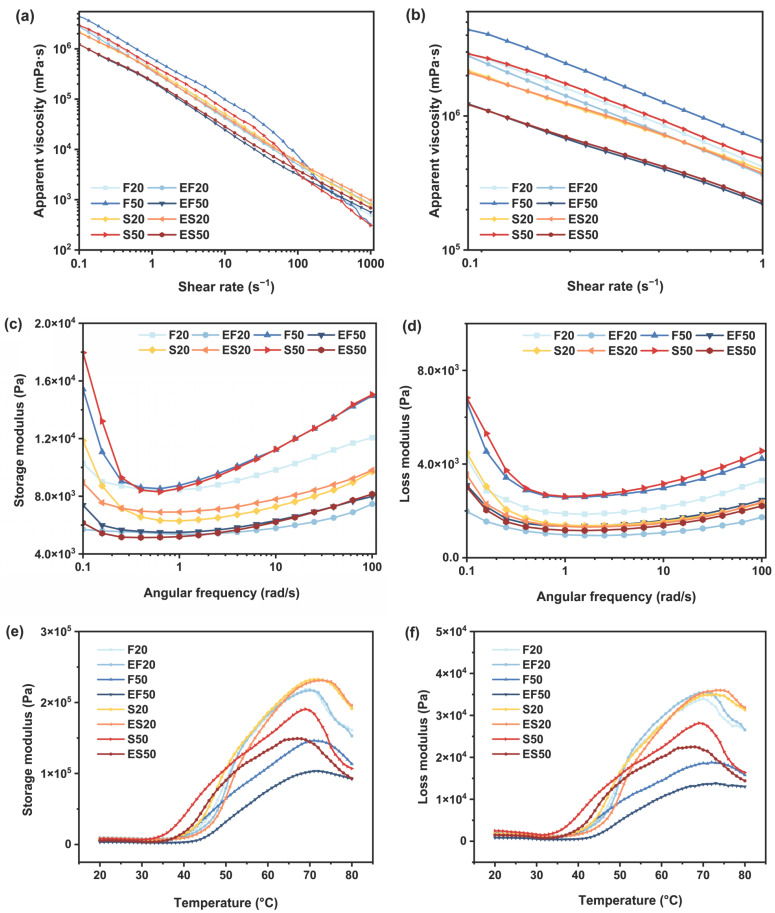
Rheological properties of emulsified meat batters from different treatment groups. (**a**,**b**) Apparent viscosity curves: (**a**) overall view; (**b**) partial magnification at low shear rates. (**c**,**d**) Dynamic frequency sweep characteristics: (**c**) storage modulus (G′); (**d**) loss modulus (G″). (**e**,**f**) Dynamic temperature sweep characteristics during heating from 20 °C to 80 °C: (**e**) storage modulus (G′); (**f**) loss modulus (G″).

Apparent viscosity reflects the internal friction or flow resistance of a meat batter and generally decreases with increasing shear rate. Figure 1a,b illustrate the changes in apparent viscosity as a function of shear rate for the different treatment groups. All groups displayed shear-thinning behavior, with apparent viscosity declining as shear rate increased [28]. At low shear rates, the effect of fat replacement on viscosity varied with fat content. In the high-fat groups, substitution with MRF pre-emulsion markedly reduced viscosity, whereas the low-fat groups showed little change. Consequently, the viscosity ranking among groups with different fat contents was altered. At high shear rates, the viscosity of the unsubstituted high-fat groups (F50 and S50) decreased abruptly around 100 s^−1^; this drop was absent after substitution, suggesting that MRF pre-emulsion improved the shear stability of the meat batter.

Figure 1c,d present the changes in storage modulus (G′) and loss modulus (G″) as a function of angular frequency. Across all groups, G′ remained higher than G″ throughout the tested frequency range without crossover, indicating that the emulsified meat batters maintained an ordered, elastic structure across varying frequency conditions. All groups showed an initial decrease in G′ followed by an increase with frequency. After fat substitution, the frequency dependence of G′ became weaker, indicating that the addition of MRF pre-emulsion reduced modulus fluctuations associated with animal fat particles and enhanced system stability.

Figure 1e,f show the evolution of G′ and G″ during heating from 20 to 80 °C. All groups exhibited broadly similar trends: moduli were relatively low initially, decreased slightly, increased sharply around 40 °C, reached a peak, and finally declined. At the end of heating, the low-fat groups exhibited distinctly higher G′ values than the high-fat groups, reflecting a firmer gel network. During the sharp increase from 40 to 70 °C, the low-fat groups exhibited a steeper rise in G′, yet the curves for substituted and unsubstituted samples remained closely aligned, with minimal differences between them. In contrast, the high-fat groups, particularly S50 and ES50, showed a more gradual increase over this temperature range, but the divergence between substituted and unsubstituted pairs was substantially greater, and G′ was markedly reduced after substitution.

#### 3.1.2. Particle Size Distribution

**Figure 2:**
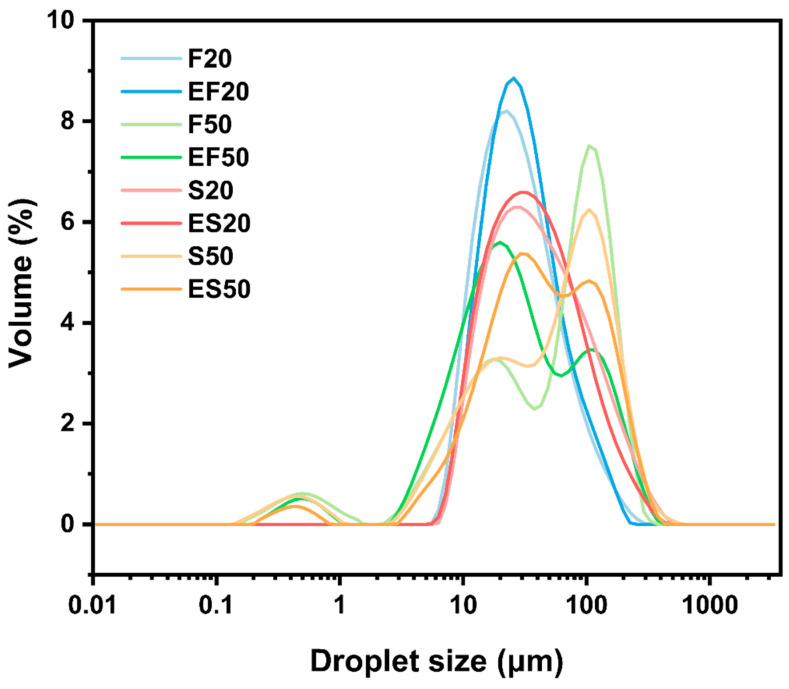
Particle size distribution of emulsified meat batters from different treatment groups.

The particle size distribution and mean droplet size of the diluted meat batters reflect the physical stability of the system. As illustrated in Figure 2, all treatment groups exhibited a main peak between 20 and 100 μm, though the distribution profiles varied with fat content. The low-fat groups showed a unimodal distribution, and the peak shifted to smaller sizes after MRF substitution. The high-fat groups exhibited a bimodal distribution, and after MRF substitution, the proportion of small particles increased, suggesting that the MRF pre-emulsion refined the fat globule size and improved emulsification stability. Across chopping intensities, the high-chopping groups displayed curves shifted leftward with higher peak values compared with their low-chopping counterparts, indicating that higher shear intensity markedly reduced particle size and improved uniformity.

Table 4 summarizes the particle size parameters for all treatment groups. D_10_, D_50_, and D_90_ correspond to the diameters at 10%, 50%, and 90% cumulative distribution, respectively; D_[3,2]_ is the surface-area-weighted mean (sensitive to small particles); and D_[4,3]_ is the volume-weighted mean (sensitive to large particles). Significant differences were observed among treatments (*p* < 0.05). High-chopping groups consistently yielded smaller droplets than low-chopping groups. High-fat groups exhibited markedly larger droplets, and the influence of chopping intensity was more pronounced in these systems. In the high-fat groups, MRF substitution significantly reduced droplet dimensions (*p* < 0.05) and lowered the proportion of large particles. Improvements were also noted in the low-chopping groups, though to a lesser extent, likely due to less effective dispersion of the pre-emulsion within the meat matrix, indicating a significant interaction between chopping intensity and formulation (*p* < 0.001). Nevertheless, these results confirm that MRF substitution provides a stabilizing effect even under milder chopping conditions. In the low-fat groups, the reduction in droplet size after substitution was modest, probably because the inherently stable low-fat system offered limited opportunity for further refinement.

#### 3.1.3. Color Analysis

Color is a crucial indicator of the appearance quality of emulsified meat products. The color parameters of raw meat batters and cooked gels are summarized in Table 5. Chopping intensity, formulation, and their interaction exerted significant effects on color parameters (*p* < 0.05). High-fat groups generally exhibited higher lightness (L*) than low-fat groups, and high-chopping groups showed higher L* values than low-chopping ones. After MRF substitution, the redness (a*) values of the low-chopping groups (ES20, ES50) decreased significantly (*p* < 0.05).

Upon heating, the batters formed dense gel structures, and lightness (L*) increased significantly (*p* < 0.05) in all groups. This increase is likely attributable to protein denaturation and the development of a compact microstructure that enhances light scattering [29]. Redness (a*) values became negative or very low after cooking. Notably, the MRF-substituted groups exhibited L* values close to or even higher than those of the unsubstituted groups, indicating that the MRF pre-emulsion effectively mimics the white, opaque appearance imparted by natural fat. Yellowness (b*) values of the substituted groups remained relatively stable, with no obvious deviation attributable to the vegetable oil component.

#### 3.1.4. Cooking Loss and Shear Force

Cooking loss and shear force results are presented in Table 5. The cooking loss and shear force were significantly influenced by chopping intensity, fat formulation, and their interaction (*p* < 0.05). Increasing fat content from 20% to 50% reduced cooking loss, indicating that added fat enhances water- and fat-holding capacity. All MRF-substituted groups exhibited higher cooking loss than their unsubstituted counterparts. Although MRF possesses good emulsifying properties, it appears less effective than natural animal fat in stabilizing the system during heat-induced gelation, resulting in greater exudation of water and oil. There was a significant interaction between chopping intensity and fat formulation (*p* < 0.001). In the high-chopping systems, however, the increase in cooking loss after substitution was relatively smaller, suggesting that high shear improves dispersion of the pre-emulsion within the matrix and partially offsets the loss.

Shear force, an indicator of tenderness and mechanical strength, is shown in Table 5. Higher fat content significantly reduced shear force (*p* < 0.05), primarily due to the melting of animal fat during heating, a key determinant of gel strength. MRF substitution reduced shear force, with a more pronounced effect observed in the low-chopping groups (S20 and S50). After substitution, the interaction between formulation and chopping intensity indicated that differences in shear force between high- and low-chopping groups became less evident, suggesting that the pre-emulsion attenuated the impact of chopping intensity on gel network strength.

#### 3.1.5. Low-Field NMR Water Distribution

**Figure 3:**
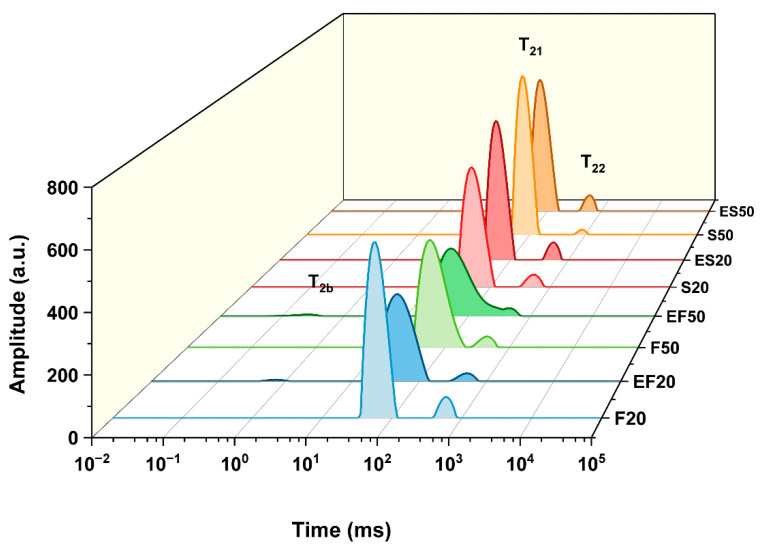
Distribution of T_2_ relaxation times in emulsified meat gels from different treatment groups measured by low-field NMR.

Low-field NMR was used to assess water distribution in the meat gels. As shown in Figure 3, all treatment groups exhibited two characteristic water peaks, with the immobilized water (T_21_) peak (20–100 ms) being the most prominent. After MRF substitution, the T_21_ peak shifted to longer relaxation times relative to the unsubstituted groups. In the ES20 group, the T_21_ peak accounted for up to 98.25% of the total signal, while the free water (T_22_) peak became larger and sharper after substitution, indicating a partial conversion of immobilized water to free water. This shift aligns with the observed increase in cooking loss. Notably, only the high-chopping substitute groups (EF20 and EF50) displayed a distinct bound water (T_2b_) signal, suggesting that high shear promotes further transformation of immobilized water into bound water and improves the overall water-binding state in MRF-containing gels.

#### 3.1.6. Texture Profile Analysis (TPA)

Texture profile analysis (TPA) parameters serve as key indicators of the quality and consumer acceptability of emulsified meat products. Table 6 summarizes the effects of the different model systems and MRF substitution on the TPA parameters of the meat gels. Two-way ANOVA revealed that chopping intensity, fat formulation, and their interaction significantly affected most TPA parameters (*p* < 0.05). In particular, hardness, gumminess, and chewiness were significantly influenced by all three effects. Under identical chopping conditions, the high-fat groups exhibited significantly lower hardness, gumminess, and chewiness than the low-fat groups (*p* < 0.05). Chopping intensity also influenced mechanical strength: low-chopping groups generally showed higher hardness than high-chopping groups. Following MRF substitution, hardness, cohesiveness, and chewiness decreased markedly, with the ES50 group displaying the lowest hardness (3731.94 ± 119.44 g). In addition, adhesiveness was significantly affected only by fat formulation (*p* = 0.005), with high-fat systems showing larger negative adhesiveness values. Notably, springiness showed an increasing trend after substitution, although no statistically significant differences were observed according to two-way ANOVA (*p* > 0.05). This textural softening reflects a distinct mode of interaction between the MRF pre-emulsion and myofibrillar proteins relative to natural fat. The presence of MRF likely altered water distribution and weakened the protein network. Nonetheless, the higher springiness values observed in high-chopping substituted systems suggest that, under intense shear, MRF and proteins may associate into a composite structure with an improved deformation recovery tendency.

### 3.2. Sensory Evaluation

#### 3.2.1. Quantitative Descriptive Analysis

**Figure 4:**
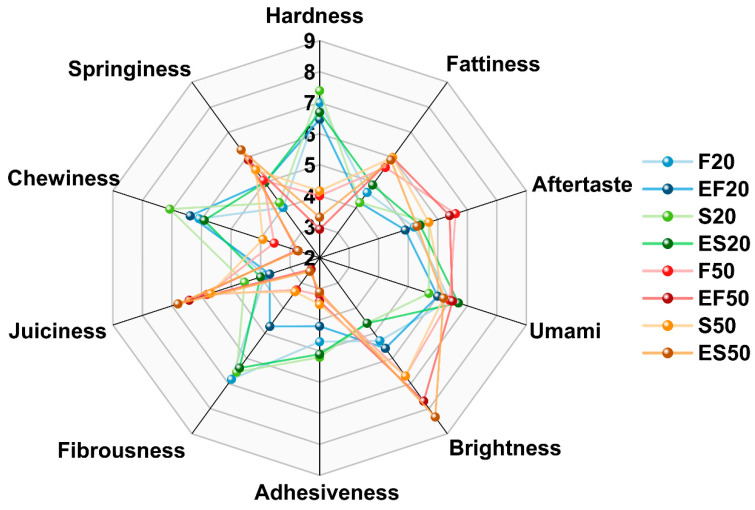
QDA scores of emulsified meat gels from different treatment groups.

Quantitative descriptive analysis (QDA) is widely used to evaluate the flavor and texture characteristics of emulsified meat products. Figure 4 presents the QDA scores for the meat gels across the different model systems and after MRF substitution. Fat content was the primary factor governing sensory quality. The low-fat groups received significantly higher scores for hardness and chewiness, whereas the high-fat groups scored higher for juiciness and fattiness, consistent with the TPA results. Regarding chopping intensity, the low-chopping groups exhibited a more pronounced fibrousness perception, while the high-chopping groups were perceived as more delicate and uniform in texture. Partial fat replacement with MRF pre-emulsion reduced perceived hardness and adhesiveness, yielding a smoother mouthfeel that alleviated the dryness and firmness typically associated with low-fat products. Importantly, the substituted groups (e.g., EF50 and ES50) retained relatively high scores for juiciness and fibrousness, thereby preserving the sensory complexity of the original full-fat formulations. Moreover, MRF substitution did not adversely affect umami or aftertaste. Collectively, these findings demonstrate that MRF substitution produces sensory profiles comparable to those of full-fat controls, enabling a substantial reduction in fat content without compromising the overall eating experience.

#### 3.2.2. Temporal Dominance of Sensations (TDS)

**Figure 5:**
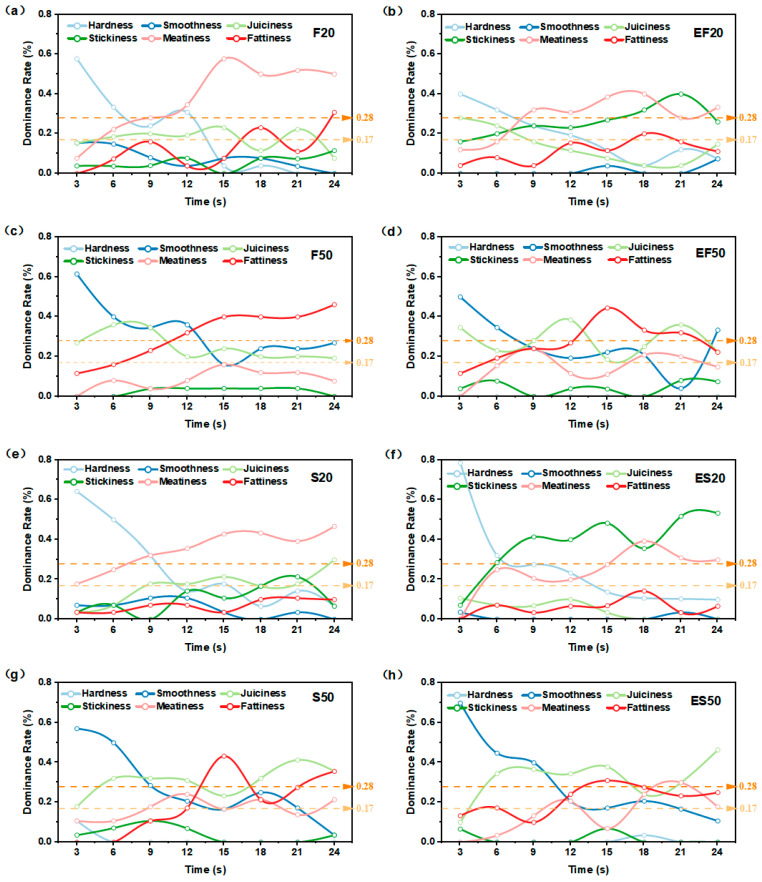
TDS curves of emulsified meat gels from different treatment groups. (**a**) F20; (**b**) EF20; (**c**) F50; (**d**) EF50; (**e**) S20; (**f**) ES20; (**g**) S50; (**h**) ES50.

TDS curves capture the dynamic evolution of sensory attributes during oral processing. In these curves, two horizontal lines denote the chance level and the significance level, respectively. The chance level corresponds to the dominance rate expected if an attribute were selected at random, whereas the significance level indicates the minimum dominance proportion required for an attribute to be considered statistically meaningful [30]. Figure 5 illustrates the dominance rate curves for six attributes (hardness, smoothness, juiciness, stickiness, meatiness, and fattiness) over a 24 s oral processing period for the different treatment groups. Comparative analysis revealed a consistent sensory progression across all treatments: an initial phase dominated by mechanical texture perception (hardness and smoothness), a middle phase characterized by structural breakdown (juiciness and stickiness), and a final phase marked by flavor release and fat perception (meatiness and fattiness).

During the early stage of oral processing (0–8 s), sensory perception was governed primarily by mechanical strength. In the low-fat groups, hardness was the dominant attribute owing to the denser gel network, whereas in the high-fat groups, smoothness prevailed, reflecting the higher fat content. These observations align closely with the dynamic temperature sweep, shear force, and texture results presented earlier. In the mid to late stages (9–16 s), as the bolus was further comminuted and mixed with saliva, the sensory profile became more complex. High-fat groups exhibited a more pronounced and sustained dominance of juiciness throughout oral processing. In the low-fat groups, fat substitution markedly increased the dominance rate of stickiness; notably, in the ES20 group, stickiness became persistently dominant from 6 s onward. In the high-fat groups, meatiness increased slightly after substitution. Regarding fat perception, MRF substitution significantly reduced the dominance rate of fattiness during the late oral processing stage (after 18 s), as seen in the EF50 and ES50 groups. This reduction effectively mitigated the greasy sensation typically associated with high-fat meat products.

Overall, the TDS curves of the fat-substituted groups closely resembled those of their respective controls, indicating that replacing 50% of pork back fat with MRF pre-emulsion successfully replicates the dynamic sensory trajectory of natural fat during oral processing. At the same time, it reduces fat perception and thereby minimizes the sensory fluctuations that often accompany fat reduction.

## 4. Discussion

### 4.1. Emulsification Stability of the Different Treatment Groups

#### 4.1.1. Rheological Properties of the Emulsified Meat Batters

Meat emulsification is a critical processing step that involves homogenizing proteins, fat, and water through chopping and shearing. Fat plays a pivotal role in stabilizing the resulting emulsion and retaining moisture. All treatment groups displayed shear-thinning behavior, consistent with shear-induced alignment of macromolecules in the meat batter along the flow direction. As reported by Choi et al., apparent viscosity is strongly influenced by both the type and amount of fat present [31]. Before substitution, the high-fat groups (F50 and S50) contained a greater number of fat globules, which increased flow resistance within the batter matrix and resulted in the highest apparent viscosity at low shear rates [32]. As the shear rate approached 100 s^−1^, viscosity declined more sharply, presumably because the batter structure was disrupted at higher shear, permitting freer movement of the fat phase. Following substitution with MRF pre-emulsion, the decrease in viscosity became more gradual and stable. This improved stability can be attributed to the water-holding capacity of MRF, which promotes a more uniform dispersed phase, reduces oil droplet coalescence, and thereby reinforces the structural integrity of the batter [33]. From a polysaccharide perspective, the long-chain structure of MRF, with its abundant hydrophilic groups, facilitates physical entanglement with myofibrillar proteins. Such entanglements not only elevate apparent viscosity at low shear rates but also buffer the disruptive effects of high shear by dissipating mechanical energy through chain rearrangement, resulting in a smoother viscosity decline [34].

Dynamic frequency sweep tests showed that, across the entire measured frequency range, G′ remained consistently higher than G″ with no crossover point, and both moduli exhibited frequency dependence, which is characteristic of weak gel rheological behavior [35,36]. For all groups, G′ initially decreased and then increased with frequency. At low frequencies, rising intermolecular friction likely reduced the elastic modulus, whereas at higher frequencies, gradual rearrangement of proteins into a more stable network contributed to the observed increase in modulus. After fat substitution, the modulus curves became flatter and less frequency-dependent, indicating that MRF pre-emulsion enhanced structural stability and dampened modulus fluctuations across the frequency range.

The effect of chopping intensity on rheological properties can be mechanistically explained by considering both the physical disruption of fat tissue and the dispersion of MRF pre-emulsion. High-speed chopping reduces fat globule size more efficiently, providing a larger specific surface area for protein adsorption, which, in the presence of MRF, promotes the formation of a more uniform protein-polysaccharide composite interfacial film [20]. This composite film exhibits weaker frequency dependence of G′, indicating enhanced structural stability. Moreover, intense shear facilitates the unfolding of myofibrillar proteins and the hydration of MRF, allowing more extensive hydrogen bonding and physical entanglement between MRF chains and proteins [21]. These interactions result in a more interconnected and shear-resistant network. From a practical standpoint, increasing chopping intensity can partially compensate for the structural weakening caused by fat reduction, especially when MRF pre-emulsion is used. Therefore, optimizing chopping intensity (e.g., 3000 r/min) is recommended for the development of low-fat emulsified meat products containing polysaccharide-based fat replacers, as it improves emulsion stability, gel strength, and texture uniformity.

#### 4.1.2. Particle Size Distribution of the Emulsified Meat Batters

Particle size distributions varied notably with fat content. The low-fat groups displayed a unimodal distribution, whereas the high-fat groups exhibited a bimodal pattern. This difference suggests that at higher fat levels, the amount of protein available per unit volume to coat fat globules is relatively limited, compromising the strength of the interfacial film. After replacing 50% of the fat with MRF pre-emulsion, the main peak shifted toward smaller diameters, and all characteristic size parameters decreased. Notably, the D_[3,2]_ value of EF50 was 83.2% lower than that of F50. It is proposed that MRF and proteins co-assemble into a composite interfacial film at the oil droplet surface, thereby enhancing resistance to coalescence. Specifically, MRF polysaccharide chains, which bear negatively charged groups (such as carboxyl and hydroxyl moieties introduced or exposed during alkaline hydrogen peroxide modification), adsorb at the oil–water interface. Through electrostatic interactions and hydrogen bonding with myofibrillar proteins, they form a thicker and more cohesive interfacial layer. This layer increases steric repulsion between adjacent droplets and suppresses droplet aggregation, thereby improving emulsion stability [37]. Li et al. similarly reported that bamboo shoot dietary fiber, when used in combination with pre-emulsification, enhanced the stability of pork batters, attributing the improvement to the strong water- and oil-holding capacities of the fiber, which promote uniform dispersion and droplet refinement [38].

### 4.2. Gel Structure Formation of the Different Treatment Groups

#### 4.2.1. Dynamic Temperature Sweep of Emulsified Meat Batters

Dynamic temperature sweep measurements track changes in the modulus of emulsified meat batters during heat-induced gelation, offering insight into the cooking behavior of these products under practical processing conditions. In the initial heating phase, G′ declined slightly, consistent with the melting of pork back fat at approximately 35 °C, where the fat transitions from a solid to a liquid state [39]. Beginning around 40 °C, the modulus started to rise as proteins underwent denaturation, and the batter gradually evolved from a weakly aggregated state into a more ordered and stable gel matrix [40]. The modulus reached a maximum near 72 °C, after which further heating caused a decline, likely reflecting excessive contraction of the protein network accompanied by the release of water and melted fat. At the end of heating, the low-fat groups displayed markedly higher G′ values than the high-fat groups. During gelation, fat globules become dispersed and embedded within the protein matrix; however, when present in excess, they cannot be fully encapsulated, leading to a less cohesive network. In contrast, systems containing MRF benefited from the thermal stability of the polysaccharide. MRF does not melt but persists as a hydrated network capable of interacting with denatured proteins through hydrogen bonding and hydrophobic associations, thereby partially offsetting the loss of structural integrity caused by fat melting [41]. The melting of back fat at elevated temperatures also contributes to the lower final G′ values observed in the high-fat systems.

#### 4.2.2. Cooking Loss

During heating of emulsified meat products, protein denaturation diminishes the capacity to retain both water and fat. As noted by Kumar, animal fat particles can function as a physical barrier that traps moisture within the protein matrix [42]. Consequently, both the water-holding capacity and cooking stability of the meat batter are strongly influenced by fat content. Reducing fat content led to a significant increase in cooking loss (*p* < 0.05), and MRF substitution likewise resulted in higher cooking loss. Previous work has shown that fats with lower melting points or greater unsaturation tend to reduce emulsion stability, contributing to yield loss and textural deterioration in products such as sausages [43]. Although MRF exhibits favorable emulsifying properties, exudation of water and oil increased during heat-induced gelation. The increase in cooking loss was less pronounced in high-chopping systems, suggesting that the composite interfacial film formed by MRF and proteins is less effective at cross-linking with the protein matrix during heating than a purely protein-based interfacial film. Similar observations were made by Pereira et al., who reported a marked rise in cooking loss when 50% of animal fat in emulsified sausages was replaced with pre-emulsified sunflower oil [44]. This apparent paradox can be resolved by considering the different thermal responses of the two components. Myofibrillar proteins undergo heat-induced denaturation and network contraction, while MRF polysaccharide chains remain thermally stable and maintain their hydrated structure. This mismatch in thermal behavior weakens the integration of the composite interfacial film into the contracting protein matrix, thereby promoting the exudation of water and oil during heating.

### 4.3. Influence of MRF Substitution on the Gel Structure of Emulsified Meat Gels

#### 4.3.1. Shear Force of Meat Gels

The shear force of the meat gels decreased following substitution with MRF pre-emulsion, consistent with the earlier discussion regarding the influence of dietary fiber on protein gel networks. Although the pre-emulsion can occupy space within the gel matrix, its interactions with proteins and fat are weaker than the covalent cross-links formed among proteins. Moreover, the pre-emulsion effectively dilutes the protein phase, yielding a less dense gel network. This shear force reduction aligns with previous observations in meat systems containing pre-emulsified oils or polysaccharide-based fat replacers, where increased water content and protein network dilution similarly resulted in lower gel strength [32,45]. This reduction in mechanical strength implies that substituted gels are more readily fragmented during mastication.

#### 4.3.2. Water Distribution

The transverse relaxation time (T_2_) determined by low-field NMR provides a sensitive measure of the distribution and mobility of different water populations within meat gels [46]. After replacing 50% of the fat with MRF pre-emulsion, the pre-emulsion droplets became dispersed throughout the protein network. Although some droplets associated with proteins to form composite interfacial films, their interactions with the surrounding matrix were comparatively weak, disrupting network continuity and reducing the physical entrapment of water. As a result, a portion of immobilized water converted to free water, consistent with the observed increase in cooking loss. These findings establish a molecular basis for the changes in macroscopic quality: the partial shift from immobilized to free water accounts for both the higher cooking loss and the reduced gel hardness.

#### 4.3.3. Texture Profile Analysis

Texture properties are fundamental quality attributes of meat products. As reported by Urgu-Öztürk et al., protein content influences gumminess, chewiness, and springiness, whereas higher fat levels tend to increase hardness and cohesiveness [47]. In the present study, MRF pre-emulsion substitution led to significantly lower hardness, cohesiveness, and chewiness relative to the unsubstituted controls. This observation aligns with the findings of Zhuang et al., who noted that replacing a portion of animal fat with pre-emulsified sesame oil in emulsified meat batters reduced TPA parameters, primarily due to the accompanying increase in water content [32]. Notably, although MRF substitution weakened the overall gel network, springiness increased. MRF exhibits a strong water-binding capacity; upon hydration and incorporation into the meat batter, the retained water distributes between fibers and proteins, allowing the system to absorb energy through deformation under applied force. The emergence of T_2b_ signals in the EF20 and EF50 groups likely contributes to this enhanced springiness. This effect can be explained by the ability of MRF polysaccharide chains to immobilize water within the gel network through hydrogen bonding, thereby raising the proportion of tightly bound water. The hydrated MRF functions as a flexible filler that deforms under stress and recovers upon unloading, thereby improving springiness without disrupting overall gel integrity [48].

### 4.4. Appearance and Sensory Quality of Emulsified Meat Gels from Different Treatment Groups

#### 4.4.1. Color

The color of meat products strongly influences consumer purchase intent and eating appeal. Consumers generally favor products with low b* values (reduced yellowness), high L* values (lightness), and elevated overall whiteness [49]. In this study, the L* values of high-fat groups were significantly higher both before and after cooking, a consequence of pronounced light scattering by abundant fat globules. High-chopping groups also displayed higher L* values, as the finer fat particles generated under intense shear are more uniformly dispersed within the protein matrix, thereby enhancing light reflection. MRF substitution markedly reduced a* (redness), attributable to the inherent color of the dietary fiber, while L* increased significantly. Notably, the L* value of EF20 surpassed that of the higher-fat EF50 group. After cooking, the L* values of MRF-substituted groups were comparable to or even exceeded those of the unsubstituted controls. These observations agree with Feng et al., who reported that substituting with dietary fiber pre-emulsions effectively preserves the desired color attributes of emulsified meat products, thereby offsetting the visual alterations typically associated with fat reduction [49].

#### 4.4.2. Quantitative Descriptive Analysis (QDA)

Quantitative descriptive analysis was employed to evaluate the sensory attributes of the emulsified meat gels. In high-fat systems, the abundant fat phase reduced the rigidity of the protein gel network, whereas the low-fat groups, with their higher relative protein content and denser networks, yielded higher hardness scores. During mastication, the gel network breaks down and releases entrapped water and fat. Low-chopping groups contained larger fat particles and more intact meat fibers, which contributed to a more pronounced fibrousness perception and a texture more reminiscent of natural meat. Conversely, high-chopping groups were perceived as more delicate and uniform, reflecting their greater structural homogeneity. Following substitution with MRF pre-emulsion, both hardness and adhesiveness decreased, and the mouthfeel became smoother. This shift is consistent with the instrumental data: the pre-emulsion disrupted the gel structure, resulting in a softer texture. At the same time, fibrousness increased slightly in the substituted groups, likely because the composite network formed by fiber and protein mimics the textural layering characteristic of natural meat during oral processing.

#### 4.4.3. Temporal Dominance of Sensations (TDS)

TDS is an advanced sensory method that captures the dominant perceptual attributes evolving over time during consumption. Texture attributes were the primary sensory features for all samples and also exhibited the greatest variation among treatments, consistent with earlier reports [50]. Owing to differences in gel architecture, the unsubstituted meat gels possessed a more continuous protein network and greater resistance to shear, whereas the fiber-induced structural modifications in the substituted groups attenuated the perception of hardness. As oral processing continued, the substituted groups retained springiness as a dominant sensation for a longer period, in agreement with their higher instrumental springiness values. Paglarini et al. observed in a study of low-salt emulsified sausages that fatty flavor is a major driver of consumer rejection [51]. In the present work, fattiness did not emerge as a notable attribute in the low-fat groups, but the high-fat groups displayed a clear dominance of fattiness during the late oral processing stage (after 18 s). MRF pre-emulsion substitution significantly reduced the dominance rate of fattiness during this period. This reduction likely arises because MRF polysaccharides, with their high water-binding capacity and interfacial activity, promote the formation of a mixed emulsion or saliva-in-oil system during mastication. Unlike free animal fat, which coalesces and coats oral surfaces, MRF-stabilized oil droplets remain finely dispersed and are cleared more readily, thereby diminishing the lingering fatty sensation.

In many studies employing vegetable oil-based fat replacers, sensory scores have declined [52,53]. For instance, Franco et al. reported that when linseed oil oleogels replaced animal fat in emulsified sausages, the overall acceptability scores for 25% and 50% substitution were 44 and 64, respectively, compared with 84 for the full-fat control [54]. In the present study, both QDA and TDS results demonstrated that the sensory profiles of MRF-substituted samples closely resembled those of the full-fat controls, with the notable exception of reduced fattiness. This indicates that the MRF pre-emulsion effectively preserves desirable sensory qualities while enabling a substantial reduction in fat content.

## 5. Conclusions

In this study, a pre-emulsion prepared with modified rice bran dietary fiber (MRF) and soybean oil was employed as a fat replacer, and four model emulsified meat gel systems were constructed by varying fat content (20% and 50%) and chopping intensity (low vs. high). The effects of MRF pre-emulsion substitution on the rheological, processing, textural, and sensory properties of both meat batters and gels were systematically evaluated. The results showed that substitution with the pre-emulsion significantly refined fat globule size and promoted more uniform fat dispersion within the meat batter, accompanied by a weaker frequency dependence of the storage modulus, indicating improved structural stability. High-chopping intensity further enhanced shear stability and structural organization. Fat content was the primary factor governing the processing and textural characteristics of the gels. High-fat systems exhibited lower cooking loss, reduced hardness and chewiness, and superior juiciness. Following MRF substitution, a partial shift from immobilized to free water was observed, which contributed to an increase in cooking loss (by 2–4 percentage points) and a reduction in hardness and chewiness, but also imparted a measurable improvement in gel springiness. A strong correspondence emerged between physicochemical properties and sensory perception. The lower hardness, lower shear force, and greater juiciness of the high-fat groups aligned closely with their higher QDA scores for juiciness and fattiness. The MRF-induced textural softening was reflected in enhanced smoothness and juiciness, as well as reduced fibrousness and hardness in the TDS profiles. Moreover, fat replacement effectively suppressed the perception of fattiness during the late stages of oral processing. In summary, MRF pre-emulsion promoted fat globule refinement, improved emulsion stability, and increased gel springiness, but also led to higher cooking loss and lower hardness. Despite these limitations, MRF-based pre-emulsions can balance structural integrity, processing stability, and dynamic sensory quality in reduced-fat emulsified meat products. Thus, MRF represents a viable polysaccharide-based fat replacer for emulsified meat products.

## Figures and Tables

**Table 1:** Representation methods of different treatment groups.

Groups	Low Chopping Intensity (S) (1500 r/min)	High Chopping Intensity (F) (3000 r/min)
Low fat content (20%)	S20	F20
High fat content (50%)	S50	F50
50% fat replacement with MRF pre-emulsion	Prefix E added to the above codes (e.g., ES20, EF20, ES50, EF50)	

**Table 2:** Formulations of emulsified meat batter samples.

Ingredient (g/500 g)	F20/S20	EF20/ES20	F50/S50	EF50/ES50
Chicken breast	320	320	200	200
Pork back fat	80	40	200	100
MRF emulsion	0	40	0	100
Sodium tripolyphosphate	1.5	1.5	1.5	1.5
Table salt	5	5	5	5
White granulated sugar	2.5	2.5	2.5	2.5
Chicken essence	1.5	1.5	1.5	1.5
White pepper powder	1.5	1.5	1.5	1.5
Ice water	88	88	88	88

Note: For each treatment group (F20/S20, EF20/ES20, F50/S50, EF50/ES50), five independent batches (*n* = 5) of emulsified meat batter were prepared and used for all subsequent laboratory analyses.

**Table 3:** Sensory attributes, definitions, and reference materials used for QDA evaluation.

Attribute	Definition	Reference
Brightness	Surface glossiness of the sample	Rice porridge = 0, fresh vegetables = 10
Hardness	Force required to deform/break the sample	Egg custard = 0, dried yak meat = 10
Springiness	Ability to recover shape after chewing	Egg yolk = 0, egg white = 10
Chewiness	Energy required to masticate the sample to a swallowable state	Egg custard = 0, glutinous rice cake = 10
Juiciness	Release of liquid/moisture sensation in the mouth	Dry bread = 0, orange = 10
Fibrousness	Presence of meat fibers in the sample	Fish ball = 0, beef jerky = 10
Adhesiveness	Stickiness of the sample during chewing	Jelly = 0, maltose = 10
Umami	Intensity of savory taste	Vegetables = 0, soy sauce = 10
Aftertaste	Residual flavor left in the mouth after swallowing	Water = 0, coffee = 10
Fattiness	Sensation of fat in the sample	Vegetables = 0, pig trotters = 10

**Table 4:** Droplet size distribution parameters of emulsified meat batters from different treatment groups.

Groups	D_[4,3]_	D_[3,2]_	Dx_(__10)_	Dx_(50)_	Dx_(90)_
F20	34.88 ± 1.24 ^g^	17.14 ± 0.48 ^e^	8.91 ± 0.17 ^f^	19.40 ± 0.56 ^g^	89.70 ± 3.10 ^f^
EF20	30.02 ± 1.24 ^h^	16.06 ± 0.27 ^f^	8.67 ± 0.14 ^f^	17.96 ± 0.27 ^g^	67.08 ± 1.74 ^h^
F50	54.52 ± 1.26 ^c^	27.84 ± 0.83 ^b^	12.52 ± 0.33 ^b^	38.98 ± 1.81 ^c^	120.40 ± 1.52 ^c^
EF50	44.52 ± 0.29 ^d^	4.69 ± 0.03 ^g^	3.16 ± 0.03 ^g^	25.70 ± 0.14 ^e^	110.80 ± 0.84 ^d^
S20	42.06 ± 1.53 ^e^	23.12 ± 0.52 ^c^	11.24 ± 0.17 ^d^	28.28 ± 0.94 ^d^	96.06 ± 3.88 ^e^
ES20	37.32 ± 0.63 ^f^	19.18 ± 0.44 ^d^	9.86 ± 0.15 ^e^	22.14 ± 0.61 ^f^	81.82 ± 2.54 ^g^
S50	68.62 ± 1.97 ^a^	28.92 ± 1.35 ^a^	11.76 ± 0.51 ^c^	55.38 ± 2.99 ^a^	147.80 ± 2.59 ^a^
ES50	63.18 ± 1.12 ^b^	29.16 ± 0.62 ^a^	13.06 ± 0.21 ^a^	45.88 ± 1.47 ^b^	129.80 ± 2.39 ^b^
Chopping intensity (*p*)	<0.001	<0.001	<0.001	<0.001	<0.001
Formulation (*p*)	<0.001	<0.001	<0.001	<0.001	<0.001
Interaction (*p*)	<0.001	<0.001	<0.001	<0.001	<0.001

Note: Two-way ANOVA revealed that chopping intensity, fat formulation, and their interaction significantly affected the particle size distribution parameters of the meat batter emulsion system (*p* < 0.001). Different superscript letters (a–h) within the same column indicate significant differences among individual treatment groups (*p* < 0.05, one-way ANOVA followed by Duncan’s test), *n* = 3; D_[4,3]_: volume-weighted mean diameter; D_[3,2]_: surface-area-weighted mean diameter; D_x(10)_, D_x(50)_, D_x(90)_: diameters at 10%, 50%, and 90% cumulative volume distribution, respectively.

**Table 5:** Color parameters (L*, a*, b*) of raw meat batters and cooked gels, cooking loss (%), and shear force (N) of emulsified meat gels from different treatment groups.

Groups	Raw Batter Color			Gel Color			Cooking Loss	Shear Force
	L*	a*	b*	L*	a*	b*		
F20	75.45 ± 0.27 ^d^	1.29 ± 0.06 ^b^	14.49 ± 0.12 ^b^	84.61 ± 0.21 ^c^	−0.30 ± 0.03 ^b^	13.50 ± 0.31 ^a^	12.55 ± 0.24 ^c^	3.68 ± 0.10 ^b^
EF20	81.74 ± 0.65 ^bc^	1.02 ± 0.01 ^c^	12.91 ± 0.58 ^c^	84.68 ± 0.38 ^c^	0.02 ± 0.03 ^a^	13.86 ± 0.32 ^a^	16.66 ± 1.11 ^b^	3.25 ± 0.11 ^c^
F50	83.37 ± 0.28 ^a^	1.54 ± 0.07 ^a^	12.10 ± 0.34 ^de^	86.89 ± 0.41 ^a^	−1.39 ± 0.05 ^e^	12.43 ± 0.07 ^b^	8.43 ± 0.32 ^e^	2.52 ± 0.16 ^e^
EF50	75.40 ± 0.27 ^d^	1.33 ± 0.03 ^b^	15.38 ± 0.20 ^a^	86.60 ± 0.24 ^ab^	−0.82 ± 0.06 ^d^	11.60 ± 0.26 ^c^	10.74 ± 1.67 ^d^	2.58 ± 0.35 ^e^
S20	75.06 ± 0.60 ^d^	0.99 ± 0.05 ^c^	14.15 ± 0.61 ^b^	83.50 ± 0.27 ^e^	−0.25 ± 0.12 ^b^	12.52 ± 0.44 ^b^	10.58 ± 0.68 ^d^	4.44 ± 0.12 ^a^
ES20	73.75 ± 0.72 ^e^	0.51 ± 0.18 ^d^	14.27 ± 0.16 ^b^	84.53 ± 0.23 ^c^	−0.25 ± 0.04 ^b^	12.65 ± 0.44 ^b^	19.24 ± 0.82 ^a^	3.30 ± 0.20 ^c^
S50	80.32 ± 0.11 ^c^	1.19 ± 0.02 ^b^	11.76 ± 0.24 ^e^	83.99 ± 0.04 ^d^	−0.70 ± 0.08 ^c^	11.53 ± 0.22 ^c^	8.25 ± 0.52 ^e^	2.94 ± 0.19 ^d^
ES50	80.99 ± 1.40 ^b^	0.37 ± 0.14 ^d^	12.72 ± 0.79 ^cd^	86.34 ± 0.30 ^b^	−0.89 ± 0.02 ^d^	11.51 ± 0.05 ^c^	12.93 ± 0.41 ^c^	2.44 ± 0.07 ^e^
Chopping Intensity (*p*)	<0.001	<0.001	0.019	<0.001	<0.001	<0.001	<0.001	0.021
Formulation (*p*)	<0.001	<0.001	<0.001	<0.001	<0.001	<0.001	<0.001	<0.001
Interaction (*p*)	<0.001	<0.001	<0.001	<0.001	<0.001	0.025	<0.001	<0.001

Note: n = 3 for cooking loss and shear force; *n* = 4 for color measurements. Different superscript letters (a–e) within the same column indicate significant differences among individual treatment groups (*p* < 0.05, one-way ANOVA followed by Duncan’s test). The *p*-values for main effects (chopping intensity, fat content) and their interaction were obtained from two-way ANOVA.

**Table 6:** TPA parameters of emulsified meat gels from different treatment groups.

Groups	Hardness (g)	Adhesiveness (g*s)	Springiness	Cohesiveness	Gumminess (g)	Chewiness (g)	Resilience
F20	6612.91 ± 108.02 ^b^	−33.53 ± 7.51 ^ab^	0.81 ± 0.04 ^c^	0.72 ± 0.01 ^bc^	4770.645 ± 96.18 ^b^	4205.02 ± 184.37 ^a^	0.32 ± 0.02 ^bc^
EF20	5709.10 ± 201.10 ^d^	−39.46 ± 14.12 ^ab^	0.87 ± 0.04 ^ab^	0.63 ± 0.02 ^f^	3590.51 ± 201.11 ^e^	3016.94 ± 149.03 ^d^	0.25 ± 0.02 ^d^
F50	5141.31 ± 93.18 ^f^	−55.87 ± 6.01 ^c^	0.87 ± 0.02 ^a^	0.73 ± 0.01 ^ab^	3744.22 ± 61.53 ^e^	3266.58 ± 118.97 ^cd^	0.34 ± 0.01 ^a^
EF50	3961.00 ± 16.99 ^g^	−44.16 ± 12.72 ^abc^	0.90 ± 0.03 ^ab^	0.71 ± 0.00 ^c^	2829.49 ± 18.34 ^f^	2505.39 ± 99.19 ^e^	0.33 ± 0.01 ^b^
S20	7324.55 ± 103.11 ^a^	−35.83 ± 10.79 ^abc^	0.91 ± 0.04 ^a^	0.70 ± 0.00 ^d^	5117.99 ± 76.27 ^a^	4514.62 ± 58.69 ^a^	0.30 ± 0.00 ^c^
ES20	6394.95 ± 199.29 ^c^	−30.13 ± 5.28 ^a^	0.86 ± 0.03 ^b^	0.65 ± 0.00 ^e^	4190.20 ± 138.07 ^c^	3732.79 ± 86.09 ^b^	0.26 ± 0.00 ^d^
S50	5287.35 ± 42.65 ^e^	−53.35 ± 5.18 ^bc^	0.89 ± 0.01 ^ab^	0.73 ± 0.01 ^a^	3863.99 ± 60.60 ^d^	3310.43 ± 193.27 ^c^	0.35 ± 0.01 ^a^
ES50	3731.94 ± 119.44 ^h^	−51.07 ± 16.78 ^abc^	0.88 ± 0.02 ^ab^	0.74 ± 0.00 ^c^	2738.04 ± 90.72 ^g^	2515.20 ± 128.43 ^e^	0.31 ± 0.00 ^c^
Chopping intensity (*p*)	<0.001	0.820	0.502	0.049	<0.001	0.001	0.275
Formulation (*p*)	<0.001	0.005	0.502	<0.001	<0.001	<0.001	<0.001
Interaction (*p*)	<0.001	0.918	0.275	<0.001	<0.001	<0.001	0.026

Note: Different superscript letters (a–h) within the same column indicate significant differences among individual treatment groups (*p* < 0.05, one-way ANOVA followed by Duncan’s test). The *p*-values for main effects (chopping intensity, fat content) and their interaction were obtained from two-way ANOVA and are shown in the lower section of the table.

## Data Availability

The original contributions presented in the study are included in the article, further inquiries can be directed to the corresponding author.

## References

[1] Li Y., Xu Z., Sun J., Zhu Y. (2023). Effects of an inulin and microcrystalline cellulose hybrid hydrogel on the short-term low temperature storage characteristics of pork sausage models. Food Hydrocoll..

[2] Zhao Y.H., Zhao X., Xu X.L. (2025). Investigating the influence of myofibrillar protein and chitosan interfacial distribution on the macroscopic characteristics of emulsions. Food Chem..

[3] Wei L., Ren Y., Huang L., Ye X., Li H., Li J., Cao J., Liu X. (2024). Quality, Thermo-Rheology, and Microstructure Characteristics of Cubic Fat Substituted Pork Patties with Composite Emulsion Gel Composed of Konjac Glucomannan and Soy Protein Isolate. Gels.

[4] Asyrul-Izhar A.B., Bakar J., Sazili A.Q., Meng G.Y., Ismail-Fitry M.R. (2023). Incorporation of Different Physical Forms of Fat Replacers in the Production of Low-Fat/Reduced-Fat Meat Products: Which is More Practical?. Food Rev. Int..

[5] Machado M., Rodriguez-Alcala L.M., Gomes A.M., Pintado M. (2023). Vegetable oils oxidation: Mechanisms, consequences and protective strategies. Food Rev. Int..

[6] Li K., Zhang M., Bhandari B., Li L., Yang C. (2020). Effect of pre-emulsified soybean oil as a fat replacer on the physical and sensory attributes of reduced-fat filling in steamed buns. J. Food Process Eng..

[7] He Q.Q., Li X.F., Liu C.X., Liu Z., Zhou S., Li Y., Ma T. (2026). Application and research progress of dietary fiber-based fat substitutes in food systems. Food Res. Int..

[8] Yang P., Liu Y.W., Liu Y., Guo H., Wu D., Gan R., Li Z., Huang Y., Gao H. (2026). Comprehensive review of fat replacers utilized in sausages: Classification, advantages, and applications. Food Chem..

[9] Gu X.Y., Mao Y.X., Liu K., Zhao Y., Zha F., Xu X., Zhao Y. (2025). Construction of gel network based on soybean dietary fiber-sturgeon myofibrillar protein: Mechanism of influence in modification treatment on gel structure and properties. Food Chem..

[10] Huang C., Blecker C., Wei X., Xie X., Li S., Chen L., Zhang D. (2024). Effects of different plant polysaccharides as fat substitutes on the gel properties, microstructure and digestion characteristics of myofibrillar protein. Food Hydrocoll..

[11] Phoon P.Y., Tan Z.L.A. (2025). Synergy between curdlan and nanofibres of cellulose and chitin and its impact on the mechanical strength of oil-rich composite structures. Int. J. Biol. Macromol..

[12] Totaro M.P., Miccolis M., De Angelis D., Natrella G., Caponio F., Summo C., Faccia M. (2025). Optimization of the Rheological Properties of Fat Replacers Based on Inulin at Different Degrees of Polymerization and Their Application in Beef Burgers. Foods.

[13] Xu Y., Qi J., Qi L.W., Liu J., Zhao X., Chisoro P., Yang P., Zhang C. (2025). Agroindustrial Waste-Derived Insoluble Dietary Fibers as Solid Fat Substitutes for Meat Emulsion Gels: Feasibility, Modification Strategies, and Dual-Filling Mechanisms. Food Bioprocess Technol..

[14] Meng X., Liu F., Xiao Y., Cao J., Wang M., Duan X. (2019). Alterations in physicochemical and functional properties of buckwheat straw insoluble dietary fiber by alkaline hydrogen peroxide treatment. Food Chem. X.

[15] Hashemi B., Assadpour E., Wang Y., Jafari S.M. (2025). Pickering emulsion gels stabilized by protein and polysaccharide-based particles: A review of stability, synthesis, applications and prospective. Adv. Colloid Interface Sci..

[16] Zhao Y., Zhang W., Zhao X., Xu X. (2025). Interfacial structure in binary polymer emulsions: Probing formation and stabilization mechanisms with advanced in situ probes. Curr. Opin. Colloid Interface Sci..

[17] Zhu X.X., Tu Y.G., Zhao Y., Wu N., Yao Y., Chen S., Wei T., Mao J., Hu X., Wang S. (2025). Emulsion gel-based fat replacers in meat products: Structured design, processing stability, oral lubricity/flavor perception, and digestive characteristics. Food Chem..

[18] Neiers F., Alkan D., Amiri-Rigi A., Brandão E., Ellouze I., Grigoriadis A., Ibrahim M.N.G., Yıldırım H.K., Künili I.E., Muradova M. (2026). Food formulation: Rheological and tribological determinants of oral processing and flavor perception. Food Res. Int..

[19] Lee J.A., Kang K.M., Kim H.Y. (2025). Changes in Physicochemical Characteristics of Goat Meat Emulsion-type Sausage According to the Ratio of Fat and Water Contents. Food Sci. Anim. Resour..

[20] Zhou L., Zhang W.A., Wang J.Y., Zhang R.Y., Zhang J. (2022). Comparison of oil-in-water emulsions prepared by ultrasound, high-pressure homogenization and high-speed homogenization. Ultrason. Sonochem..

[21] Wang Y., Zhang H., Tao Y., Xu X., Zhao X. (2024). Pre-emulsion constructed with modified rice bran fiber and its application in low-fat chicken meatballs. LWT.

[22] Zhang G., Bi X., Li L., Zheng Y., Zheng D., Peng X., Jia N., Liu D. (2023). Catechins affect the oil-holding capacity of meat batters by changing the structure and emulsifying properties of surface proteins at the fat globules. Int. J. Biol. Macromol..

[23] Wang B., Chen J., Wang Q., Zhu Y., Wang P., Xu X. (2023). Functional performance of a novel emulsion gel-based pork fat mimics in low-fat meat batter system: Incorporation of physicochemical and oral processing. Food Struct..

[24] Tao Y., Cai J., Wang P., Chen J., Zhou L., Wang Q., Li Z., Wang J., Xu X. (2025). Effect of heat treatment on the stability of ultrasound-assisted cross-linked myofibrillar protein-stabilized emulsion filler phases via rheology and tribology. Food Hydrocoll..

[25] Zhao Y.H., Xu X.L., Zhao X. (2026). Mechanisms underlying the regulation of oil/water interface behavior by interfacial distribution of myofibrillar proteins and chitosan. Food Hydrocoll..

[26] Lin W., Barbut S. (2024). Hybrid meat batter system: Effects of plant proteins (pea, brown rice, faba bean) and concentrations (3–12%) on texture, microstructure, rheology, water binding, and color. Poult. Sci..

[27] Bibat M.A.D., Ang M.J., Eun J.-B. (2022). Impact of replacing pork backfat with rapeseed oleosomes—Natural pre-emulsified oil—On technological properties of meat model systems. Meat Sci..

[28] Qiu R., Wang G., Zhao P., Liu L., Awais M., Fan B., Huang Y., Tong L., Wang L., Accoroni C. (2024). Modification of the texture of 3D printing soy protein isolate-based foods with proper nozzle sizes: A swallowing oriented strategy for dysphagia diet. Int. J. Biol. Macromol..

[29] Zhu X., Zhang J., Liu S., Gu Y., Yu X., Gao F., Wang R. (2022). Relationship between Molecular Structure and Heat-Induced Gel Properties of Duck Myofibrillar Proteins Affected by the Addition of Pea Protein Isolate. Foods.

[30] Pineau N., Schlich P., Cordelle S., Mathonnière C., Issanchou S., Imbert A., Rogeaux M., Etiévant P., Köster E. (2009). Temporal Dominance of Sensations: Construction of the TDS curves and comparison with time-intensity. Food Qual. Prefer..

[31] Choi Y.-S., Kim Y.-B., Kim H.-W., Hwang K.-E., Song D.-H., Jeong T.-J., Park J., Kim C.-J. (2015). Emulsion Mapping in Pork Meat Emulsion Systems with Various Lipid Types and Brown Rice Fiber. Korean J. Food Sci. Anim. Resour..

[32] Zhuang X., Han M., Kang Z.-L., Wang K., Bai Y., Xu X.-L., Zhou G.-H. (2016). Effects of the sugarcane dietary fiber and pre-emulsified sesame oil on low-fat meat batter physicochemical property, texture, and microstructure. Meat Sci..

[33] Ağar B., Gençcelep H., Saricaoğlu F.T., Turhan S. (2016). Effect of sugar beet fiber concentrations on rheological properties of meat emulsions and their correlation with texture profile analysis. Food Bioprod. Process..

[34] Wu Y.Y., Wu Y.M., Zhao Y.Q., Xiang H., Hao Z., Wang Q., Wang Y. (2025). Enhanced stability and rheological properties of myofibrillar proteins emulsions conferred by oat β-glucan: Insights into structural and interfacial interactions. Food Chem..

[35] Cao Y., Mezzenga R. (2020). Design principles of food gels. Nat. Food.

[36] Gabriele D., de Cindio B., D’Antona P. (2001). A weak gel model for foods. Rheol. Acta.

[37] Khoder R.M., Abou-Elsoud M., Iqbal N., Khalifa I., You J., Huang Q., Yin T., Liu R. (2026). Food byproduct-derived as sustainable nano-stabilizers for Pickering emulsions influenced by macronutrient composition. Food Chem..

[38] Li K., Liu J.-Y., Fu L., Zhao Y.-Y., Zhu H., Zhang Y.-Y., Zhang H., Bai Y.-H. (2020). Effect of bamboo shoot dietary fiber on gel properties, microstructure and water distribution of pork meat batters. Asian-Australas. J. Anim. Sci..

[39] Kim T.-K., Lee M.H., Yong H.I., Jang H.W., Jung S., Choi Y.-S. (2021). Impacts of fat types and myofibrillar protein on the rheological properties and thermal stability of meat emulsion systems. Food Chem..

[40] Paglarini C.D.S., Furtado G.D.F., Honorio A.R., Mokarzel L., da Silva Vidal V.A., Ribeiro A.P.B., Cunha R.L., Pollonio M.A.R. (2019). Functional emulsion gels as pork back fat replacers in Bologna sausage. Food Struct..

[41] Zhuang X.B., Wang L.J., Jiang X.P., Chen Y.J., Zhou G.H. (2020). The effects of three polysaccharides on the gelation properties of myofibrillar protein: Phase behaviour and moisture stability. Meat Sci..

[42] Kumar Y. (2021). Development of Low-Fat/Reduced-Fat Processed Meat Products using Fat Replacers and Analogues. Food Rev. Int..

[43] Faridah M.R., Yusoff M.M., Rozzamri A., Ibadullah W.Z.W., Hairi A.N.A., Abu Daud N.H., Huda N., Ismail-Fitry M.R. (2023). Effect of Palm-Based Shortenings of Various Melting Ranges as Animal Fat Replacers on the Physicochemical Properties and Emulsion Stability of Chicken Meat Emulsion. Foods.

[44] Pereira J., Brohi S.A., Malairaj S., Zhang W.G., Zhou G.H. (2020). Quality of fat-reduced frankfurter formulated with unripe banana by-products and pre-emulsified sunflower oil. Int. J. Food Prop..

[45] Liu X., Ma Y.L., Wang L., Liu W., Wei J., Zhang C., Zhang Y., Wang W. (2025). Multifunctional bigels from beeswax and sesbania gum-gelatin: Toward natural fat replacement. LWT.

[46] Zhao S., Yang L., Chen X., Zhao Y., Ma H., Wang H., Su A. (2024). Modulation of the conformation, water distribution, and rheological properties of low-salt porcine myofibrillar protein gel influenced by modified quinoa protein. Food Chem..

[47] Urgu-Öztürk M., Öztürk-Kerimoğlu B., Serdaroğlu M. (2020). Design of healthier beef sausage formulations by hazelnut-based pre-emulsion systems as fat substitutes. Meat Sci..

[48] Feng R., Liu X.-Y., Xu B.-C., Zhang B. (2025). Evaluation of Emulsion Gel Stabilized by Konjac Glucomannan as a Pork Fat Substitute: Effect on Microstructure, Water Distribution, and Oxidative Stability of Meat Patties. J. Food Sci..

[49] Zhang H., Xiong Y., Bakry A.M., Xiong S., Yin T., Zhang B., Huang J., Liu Z., Huang Q. (2019). Effect of yeast β-glucan on gel properties, spatial structure and sensory characteristics of silver carp surimi. Food Hydrocoll..

[50] Watanabe G., Ohmori H., Tajima K., Sasaki Y., Wakiya Y., Motoyama M., Nakajima I., Sasaki K. (2019). Relative contribution of sensory characteristics for different types of pork loin, assessed by temporal dominance of sensations. J. Sci. Food Agric..

[51] Paglarini C.D.S., Silva Vidal V.A., dos Santos M., Coimbra L.O., Esmerino E.A., Cruz A.G., Pollonio M.A.R. (2020). Using dynamic sensory techniques to determine drivers of liking in sodium and fat-reduced Bologna sausage containing functional emulsion gels. Food Res. Int..

[52] Bilska A., Krzywdzińska-Bartkowiak M. (2025). The Influence of Vegetable Oil Addition Levels on the Fatty Acid Profile and Oxidative Transformation Dynamics in Liver Sausage-Type Processed Meats. Foods.

[53] Lee H.-J., Jung E.-H., Lee S.-H., Kim J.-H., Lee J.-J., Choi Y.-I. (2015). Effect of Replacing Pork Fat with Vegetable Oils on Quality Properties of Emulsion-type Pork Sausages. Korean J. Food Sci. Anim. Resour..

[54] Franco D., Martins A.J., Lopez-Pedrouso M., Purriños L., Cerqueira M.A., Vicente A.A., Pastrana L.M., Zapata C., Lorenzo J.M. (2019). Strategy towards Replacing Pork Backfat with a Linseed Oleogel in Frankfurter Sausages and Its Evaluation on Physicochemical, Nutritional, and Sensory Characteristics. Foods.

